# Gait analysis before and after corrective osteotomy in patients with knee osteoarthritis and a valgus deformity

**DOI:** 10.1007/s00167-016-4045-x

**Published:** 2016-02-18

**Authors:** N. van Egmond, N. Stolwijk, R. van Heerwaarden, A. van Kampen, N. L. W. Keijsers

**Affiliations:** 1 Department of Orthopaedics, Radboud University Medical Centre, P.O. Box 9101, 6500 HB Nijmegen, The Netherlands; 2 Institute of Health Studies, HAN University, Kapittelweg 33, 6525 EN Nijmegen, The Netherlands; 3 Department of Orthopaedics, Sint Maartenskliniek, P.O. Box 9011, 6500 GM Nijmegen, The Netherlands; 4 Research Department, Sint Maartenskliniek, P.O. Box 9011, 6500 GM Nijmegen, The Netherlands

**Keywords:** Double osteotomy, Supracondylar femoral osteotomy, Closed wedge medial high tibial osteotomy, Valgus alignment, Gait analysis

## Abstract

**Purpose:**

In this prospective study, the changes in kinetics and kinematics of gait and clinical outcomes after a varus osteotomy (tibial, femoral or double osteotomy) in patients with osteoarthritis (OA) of the knee and a valgus leg alignment were analysed and compared to healthy subjects.

**Methods:**

Twelve patients and ten healthy controls were included. Both kinetics and kinematics of gait and clinical and radiographic outcomes were evaluated.

**Results:**

The knee adduction moment increased significantly postoperatively (*p* < 0.05) and almost similar to the control group. Patients showed less knee and hip flexion/extension motion and moment during gait pre- and postoperatively compared to the controls. A significant improvement was found in WOMAC [80.8 (SD 16.1), *p* = 0.000], KOS [74.9 (SD 14.7), *p* = 0.018], OKS [21.2 (SD 7.5), *p* = 0.000] and VAS-pain [32.9 (SD 20.9), *p* = 0.003] in all patients irrespective of the osteotomy technique used. The radiographic measurements showed a mean hip knee ankle (HKA) angle correction of 10.4° (95 % CI 6.4°–14.4°).

**Conclusion:**

In patients with knee OA combined with a valgus leg alignment, the varus-producing osteotomy is a successful treatment. Postoperatively, the patients showed kinetics and kinematics of gait similar as that of a healthy control group. A significant increase in the knee adduction moment during stance phase was found, which was related to the degree of correction. The HKA angle towards zero degrees caused a medial shift in the dynamic knee loading. The medial shift will optimally restore cartilage loading forces and knee ligament balance and reduces progression of OA or the risk of OA. A significant improvement in all clinical outcomes was also found.

**Level of evidence:**

III.

## Introduction

Malalignment of the leg increases the risk of progression of knee osteoarthritis (OA) and causes a decline in physical function and progression of pain [15, 32]. One of the possible reasons for this increased risk of OA is that a malalignment of the knee influences the forces and moments acting on the knee during walking. In patients with medial knee OA and a varus alignment, an increased knee adduction moment is typically observed [17, 35, 36]. Kaufman et al. [17] found a significant difference between patients with knee OA (0.39 % BW–HT, SD 0.28) and healthy subjects (0.36 % BW–HT, SD 0.36). Turcot et al. [35] found a significant difference between patients with a varus leg alignment (0.62 Nm/kg, SD 0.19) compared to the control group (0.50 Nm/kg, SD 0.12). Moreover, the literature has shown a relationship between the degree of knee deformity and the forces acting on the knee [32, 33, 35, 36, 39, 40]. Weidenhielm et al. [39] found correlations between the hip knee ankle (HKA) angle and the peak adduction moment before surgery, after surgery and between the change in HKA angle and the change in peak adduction moment after surgery. Furthermore, varus alignment and increased knee adduction moment were associated with the progression of OA [25, 26, 32]. Sharma et al. [32] found a significant correlation between adduction moment and the Kellgren–Lawrence grade in knees. They also found significant correlations between adduction moment and joint space width in knees. In another study, Sharma and Song [33] found that a varus alignment was associated with a fourfold increase in the odds of medial progression (adjusted odds ratio 4.09, 95 % CI 2.20–7.62). Hence, malalignment of the leg alters the kinetics and kinematics in the knee, which most likely increases the risk of knee OA [32, 35].

**Fig. 1:**
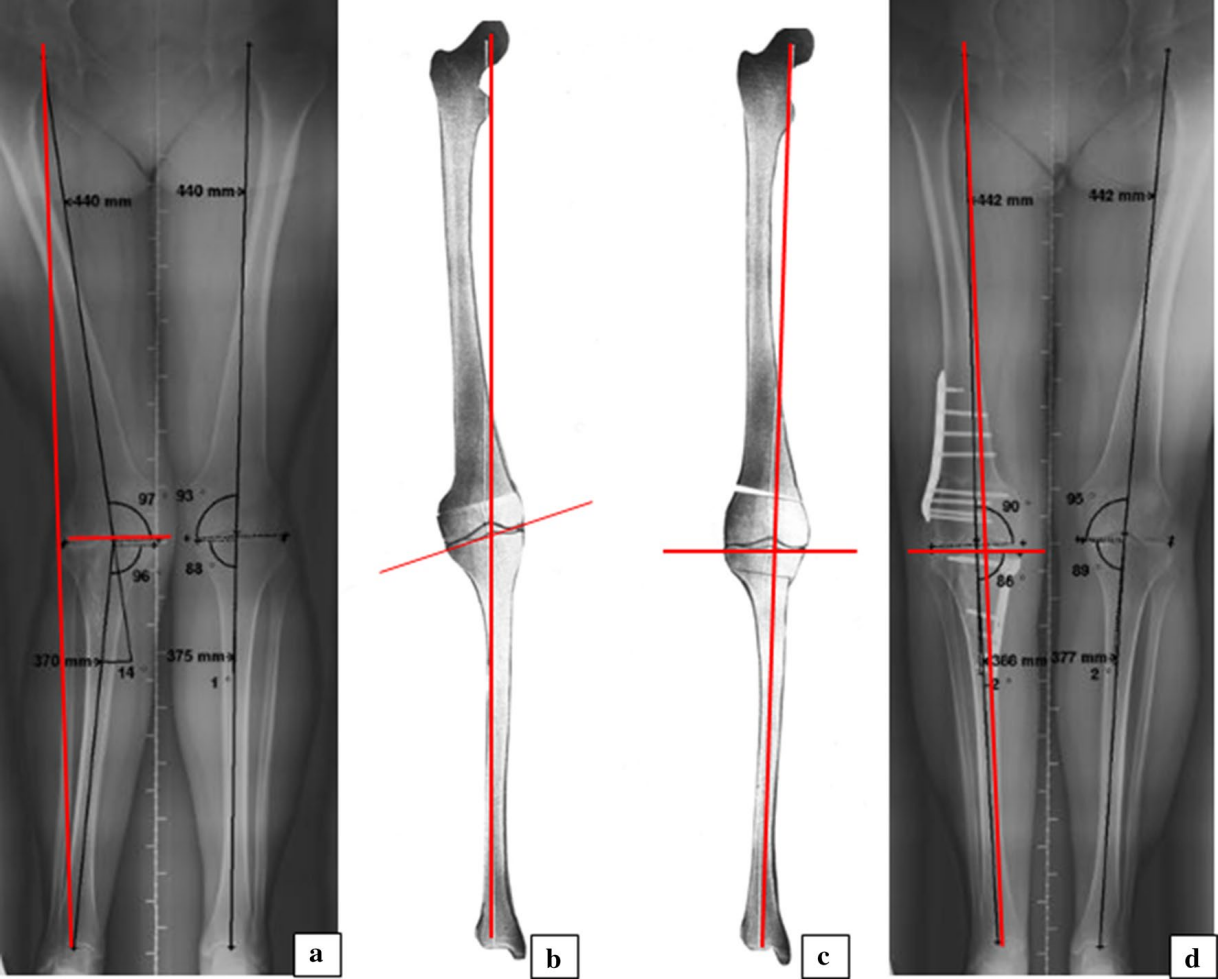
Rationale of double osteotomy in valgus corrective surgery. Weight-bearing long-leg radiographs and planning drawings including weight-bearing lines (WBL) and knee joint orientation lines (KJOL) of one of the study patients. **a** Preoperative valgus leg alignment caused by femoral and tibial bone deformity, WBL lateral and KJOL neutral. **b** Planning drawing of medial closing wedge distal femur osteotomy resulting in neutral WBL and severe valgus KJOL. **c** Planning drawing of double osteotomy, i.e. lateral open wedge distal femur and medial closing proximal tibial osteotomy, resulting in neutral WBL and neutral KJOL. **d** Postoperative leg alignment after double osteotomy

When conservative treatment is no longer successful, corrective osteotomy is considered for young and active patients with lateral knee OA and a valgus leg alignment [15]. The purpose of a correction osteotomy is to realign the weight-bearing lines while maintaining normal knee joint line orientation (Fig. 1) [1, 2, 6, 7, 9–11, 14, 22, 23, 27, 28, 30, 34, 37]. A corrective osteotomy can be performed in either the femur or tibia or in both bones, i.e. a double osteotomy.

The kinetic and kinematics of gait of a varus medial osteoarthritic knee and the effect of a valgus osteotomy on these gait characteristics are well described in the literature [8, 17, 21, 25, 35, 36–39]. It is proven that a valgus-producing osteotomy is able to improve the kinetics and kinematics of gait [21, 38], causing improvements in clinical results and quality of life [4, 14]. Lind et al. [21] found a significant increase in walking speed, maximum knee flexion and a significant decrease in the mean maximum adduction moment after a valgus-producing osteotomy. Some literature addressed that the amount of adduction moment is a predictive value for the clinical results after a valgus osteotomy. Patients with a higher adduction moment showed inferior clinical results compared to patients with a lower adduction moment [29, 38]. Also, the improvements in kinetics and kinematics of gait following a valgus osteotomy decrease the rate of the progression of medial knee OA, thereby delaying or preventing later conversion to a knee arthroplasty.

Although the kinetics and kinematics of gait in a medial varus osteoarthritic knee and the effect of a valgus osteotomy are well described [8, 36, 38, 39], the effect of a varus osteotomy on gait has been investigated only once [6]. In that study, only one parameter, the knee peak adduction moment, was studied in a subgroup of 12 patients with a lateral open wedge high tibial osteotomy and a mild valgus malalignment [mean HKA angle 2.4° (SD 2.4)] without an abnormal mechanical lateral distal femoral angle (mLDFA). The authors found a significant increase in the peak knee adduction moment during gait (mean change (95 % CI) of 0.72 % BW*Ht (0.42, 1.02) suggesting a medial shift in dynamic knee joint load. Although the peak adduction moment is an important outcome, it is a simplification of describing the effect of a varus osteotomy on gait. Detailed kinetics and kinematics of gait after a varus osteotomy have not yet been described in the literature. The spatiotemporal parameters, the flexion/extension angles, the abduction/adduction angles, the flexion/extension moments, the abduction/adduction moments of the knee and hip during the whole stance phase are important parameters in gait studies [24]. Furthermore, in contrast to the study of Collins et al. [6], patients with a large HKA angle and with an abnormal mLDFA were included. As a consequence, patients who underwent a medial closing wedge high tibial osteotomy (TKO), a medial closing wedge distal femur osteotomy (SCO) or both double osteotomy (DOT) were included. Clinical results after a varus-producing osteotomy are somewhat better described, but there is a lot of discrepancy between these studies and most studies have a low level of evidence [1, 2, 7, 10, 11, 30, 34]. Therefore, a well-performed study with a complete analysis of kinetics and kinematics of gait in combination with valid clinical scores is necessary.

The purpose of the study was to evaluate changes in gait and clinical outcomes after a varus-producing osteotomy in patients with lateral OA of the knee and a valgus leg alignment and compare these to a normal control group. Based on the previous study of Collins et al. [6], who found a significant increase in knee peak adduction moment, an increase in knee adduction moment during the whole stance phase was expected. We hypothesized that all the kinetics and kinematics of gait will improve towards that of a healthy control group postoperatively due to a correction of the valgus malalignment towards a varus alignment. Such an improvement will optimally restore cartilage loading forces and knee ligament balance and reduces progression of OA or the risk of OA. An improvement in the clinical outcomes postoperative was expected.

## Materials and methods

This prospective study was carried out between 2006 and 2008, after approval of the Medical Ethical Board/Committee without an assigned number, as this study was in line with our normal protocol for operating these patients. A consecutive series of 12 patients participated in this study. Patients had been indicated for a single-level or double-level varus osteotomy because of lateral OA of the knee and a valgus alignment. Exclusion criteria were conditions other than the OA of the knee that severely influenced gait. Ten healthy control subjects participated in the study. Written informed consent was obtained prior to participation. Patients were tested preoperatively (baseline) and 1 year postoperative, whereas control subjects were only measured once. Patient characteristics at baseline and controls characteristics are presented in Table 1.

**Table 1:** Baseline parameters

Parameter	Patients	Controls
Number of subjects	12	10
Age [years (SD)]	45 (3.3)	51 (13.2)
Sex (*N*)		
Female	8	6
Male	4	4
Height [cm (SD)]	176 (13)	174 (12)
Weight [kg (SD)]	81 (14.0)	76 (8.9)
Side (Left/Right)	5/7	
OA classification (SD)		
Medial	1.4 (0.8)^a^	
Lateral	2.3 (1.1)^a^	

*SD* standard deviation, *N* number, *cm* centimetre, *kg* kilogram, *OA* osteoarthritis classification Kellgren and Lawrence

^a^ *N* = 11

### Operation techniques

Deformity analysis according to Paley and Pfeil [28] revealed a single-level femoral valgus deformity in five patients, single-level tibial valgus deformity in three patients and double-level valgus deformity in four patients. Planning of deformity correction was aimed at correction of the lower leg to a neutral mechanical axis by angular correction of the deformed bone(s) to normal or into slight varus taking care of normal knee joint orientation (Fig. 1). All osteotomies were uniplanar closing wedge corrections, which were performed by one surgeon (RvH), five medial closing wedge distal femur osteotomies (SCO), three medial closing wedge high tibial osteotomies (TKO) and a combination of both in the four double osteotomy patients (DOT). Preoperatively, a calibrated sawguide including a goniometer was used to enable accurate wedge resections according to the preoperative planning [22]. All osteotomies were fixed with angular stable (TomoFix^®^) plates. Postoperative rehabilitation consisted of immediate range of motion exercises, muscle training and partial weight-bearing until 6 weeks postoperative. Subsequently, full weight-bearing was started depending on pain and radiographic proof of sufficient bone healing.

### Clinical and radiographic outcomes

The clinical evaluation consisted of the Visual Analogue Scale for maximum pain (VAS-pm) and the frequency (VAS-pf) the patient experienced pain, The Dutch Western Ontario and McMaster Universities osteoarthritis index (WOMAC) [31], the Oxford Knee Score (OKS) [13], the Knee Outcome Survey Activities of Daily Living Scale (KOS) [16] and an evaluation of postoperative complications and reoperations. Whole leg standing anteroposterior radiographs were used to measure the pre- and postoperative hip knee ankle (HKA) angle, mechanical lateral distal femoral angle (mLDFA) and medial proximal femoral angle (MPTA), according to Paley and Pfeil [28]. Radiographic OA grading of the affected knee was performed by an independent investigator (NvE) using the Kellgren and Lawrence classification [18].

### Gait analysis methods

The kinetics and kinematics of gait of each subject were measured using the Vicon motion analysis system (Vicon Motion Systems Ltd., Oxford, UK). The study of Koenraadt et al. [20] showed an accuracy of the system of at least 0.1 mm. The system consisted of eight infrared cameras and a computer system for data acquisition, processing and analysis. Marker positions were sampled at 200 Hz. Twenty reflective markers (14 mm in diameter) were placed according to the Helen Hayes lower limb model. Kinetic data were obtained simultaneously with the measurement of the kinematics using a Kistler force plate (Kistler Instruments, Switzerland) embedded in the floor and sampling at 2400 Hz. All subjects were instructed to walk barefoot at a self-selected speed. Subjects had a fixed starting point so that their third step was placed on the surface of the force plate [5]. At least three acceptable trials were obtained for both the right and the left leg. The gait data were processed using Vicon Workstation (version 5.2) and the Optimized Lower limb Gait Analysis (OLGA) model. A Woltering filtering routine with MSE = 25 was used to filter the data.

The gait parameters of interest were walking speed, stride length and foot progression angle. In addition, varus/valgus (adduction/abduction) and flexion/extension angle and external moment of the knee and hip during the entire stance phase were obtained and subsequently normalized to stance time. Heel strike and toe-off were determined using the vertical ground reaction force with a threshold of 10 N. The average of three trials per subject was used. For each OA patient, these parameters were calculated for the affected leg, whereas for the control group, the leg was randomly selected. The kinetics and kinematics of gait were analysed using custom written programs in Matlab. The accuracy of the used method in assessing the kinematics and kinetics of gait is <5 degrees as has been reported in the literature [24, 41].

### Statistical analysis

Total test scores [mean, standard deviation (SD)] for the continuous variables (HKA angle, WOMAC, KSS, OKS, KOS, VAS-pm, VAS-pf) were calculated at baseline (preoperative) and 1 year postoperative. A paired *t* test was used to indicate differences between the preoperative and postoperative clinical outcomes and the gait characteristics walking velocity, stride length and foot progression angle.

A Wilcoxon signed-rank test was used to test for significant differences in knee and hip angles and moments between the pre- and postoperative condition for each per cent of the stance phase. Differences between the patients and controls were tested using a Mann–Whitney *U* test. To study the relation between the degree of deformity correction with the knee adduction moment, the mean knee adduction moment over the stance phase was first calculated. Subsequently, the Pearson correlation coefficient between the correction of HKA angle and increase of mean knee adduction moment was calculated. The effect of the three different surgical interventions on kinetics and kinematics were also analysed. However, no statistical analysis has been performed on these data because the subgroups consisted of only a few patients. A *p* < 0.05 was considered significant. All data were statistically analysed with SPSS version 18.0.

## Results

Not all patients had a complete data set. One patient had no preoperative clinical measurements and was therefore left out in the analysis of clinical outcomes. Two patients had an incomplete radiographic file and were therefore left out in the radiographic analysis.

### Clinical and radiographic outcomes, complications and reoperations

Postoperative all clinical results significantly improved (Table 2). The radiographic measurements showed a mean HKA angle correction of 10.4° (95 % CI 6.4°–14.4°). The mean mLDFA and MPTA are also shown in Table 2. In five patients (three DOT and two SCO), the hardware was removed within 1 year. One patient (SCO) underwent a pseudoarthrosis repair 6 months postoperative. No intraoperative or postoperative complications that could interfere with postoperative gait were found.

**Table 2:** Clinical and radiographic outcomes

Parameter	Mean preoperative scores (SD) *N* = 11	Mean postoperative scores (SD) *N* = 12	Mean difference (95 % CI) *N* = 11	*p* value *N* = 11
HKA (°)	9.3 (5.7) valgus^b^	1.1 (2.3) varus^a^	10.4 (6.4–14.4)	*p* = 0.000
mLDFA (°)	85.0 (6.2)^b^	90.0 (2.0)^a^	4.0 (0.1–7.9)	n.s.
MPTA (°)	88.0 (7.7)^b^	89.0 (2.8)^a^	0.0 (−4.4 to 4.4)	n.s.
WOMAC (0–96)	57 (13)	81 (16)	−26 (−33 to −19)	*p* = 0.000
VAS-pm (0–100)	60 (19)	33 (21)	26 (11–41)	*p* = 0.003
VAS-pf (0–100)	71 (21)	27 (22)	26 (61)	*p* = 0.000
KOS (0–100 %)	56 (15)	75 (15)	20 (35–4.2)	*p* = 0.018
OKS (12–60)	33 (8)	21 (8)	12 (6.8–17)	*p* = 0.000

*SD* standard deviation, *CI* confidence interval, *N* number, *OA* osteoarthritis classification Kellgren and Lawrence, *HKA* hip knee ankle angle, *mLDFA* mechanical lateral distal femoral angle, *MPTA* medial proximal tibial angle, *WOMAC* Dutch Western Ontario and McMaster Universities osteoarthritis index, *VAS*-*pm* Visual Analogue Scale for maximum pain, *VAS*-*pf* Visual Analogue Scale for how frequent the patient experienced pain, *KOS* Knee Outcome Survey Activities of Daily Living Scale, *OKS* Oxford Knee Score

^a^ *N* = 10

^b^ *N* = 12

### Gait analysis

#### Spatiotemporal parameters

The spatiotemporal parameters are shown in Table 3. Surgery did not affect the walking velocity of the patients, leaving a significant difference with the control group after surgery (*p* = 0.024). Although the stride length did not increase postoperatively, stride length after surgery was not significantly different from controls (*p* = 0.13). There was also no significant difference in foot progression angle between the preoperative, postoperative measurements and the controls.

**Table 3:** Spatiotemporal parameters

Spatiotemporal parameters	Mean preoperative scores (SD) *N* = 12	Mean scores control group (SD) *N* = 10	*p* value^a^	Mean postoperative scores (SD) *N* = 12	*p* value^b^
Walking velocity (m/s)	0.95 (0.09)	1.25 (0.15)	*p* < 0.001	0.93 (0.25)	n.s.
Stride length (m)	1.10 (0.18)	1.38 (0.14)	*p* = 0.004	1.17 (0.39)	n.s.
Foot progression angle (°)	7.0° (3.8°)	6.4° (2.5°)	n.s.	5.8° (3.6°)	n.s.

*SD* standard deviation, *N* number *n.s.* nonsignificant

^a^Difference between preoperative scores and scores control group

^b^Difference between preoperative and postoperative scores

#### Knee and hip kinematics

**Fig. 2:**
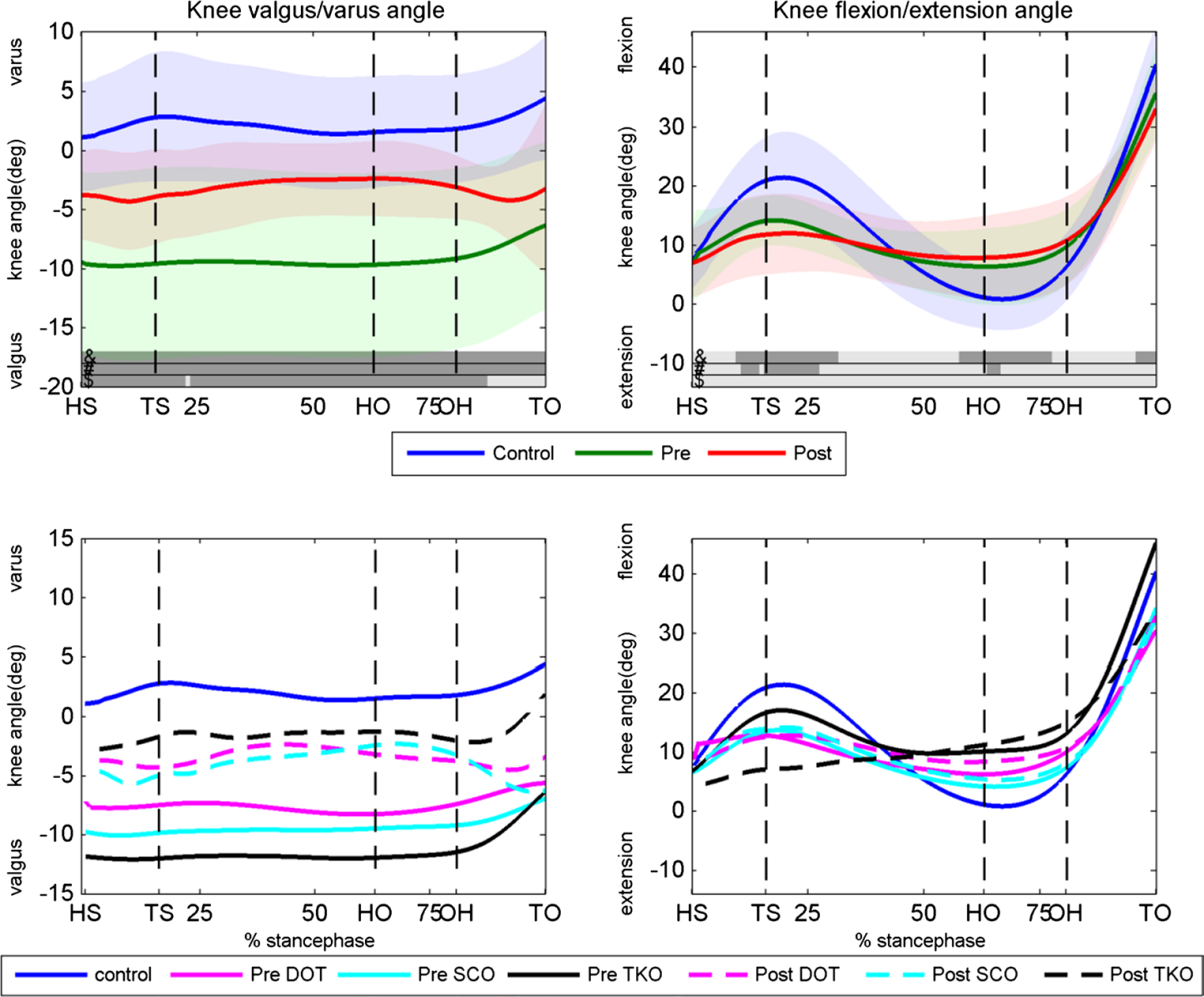
Knee valgus/varus and flexion/extension angles. *Upper panels* the knee angles of the controls, preoperative condition and postoperative condition for the valgus/varus (*left panel*) and flexion/extension (*right panel*) angle. Dark areas in the *bars right* above the *x*-axis indicate significant differences (*p* < 0.05) between: &, postoperative and controls; #, preoperative and controls; $, pre- and postoperative. *Lower panels* knee varus/valgus and flexion/extension angles for the DOT, SCO and TKO group. Pre- and postoperative as well as the control data are displayed. *HS* heel strike, *TS* toe strike, *HO* heel off, *OH* opposite heel strike, *TO* toe-off, *Deg* degrees, *Pre* preoperative, *Post* postoperative, *DOT* double osteotomy, *SCO* supracondylar osteotomy, *TKO* high tibial osteotomy

The valgus/varus and flexion/extension angle of the knee for the preoperative condition, the postoperative condition and the control group are shown in Fig. 2. Although the valgus angle of the patients significantly decreased postoperatively (except for late stance), patients had significantly more knee valgus angle during the entire stance phase before and after surgery compared to healthy controls.

Patients had pre- and postoperatively significantly less knee flexion around toe strike and less knee extension around heel off compared to controls. Knee flexion/extension angle was not significantly different between pre- and postoperative. The three types of surgery (TKO, SCO and DOT) influenced the knee angles in almost a similar manner as can be seen in the lower panels of Fig. 2.

**Fig. 3:**
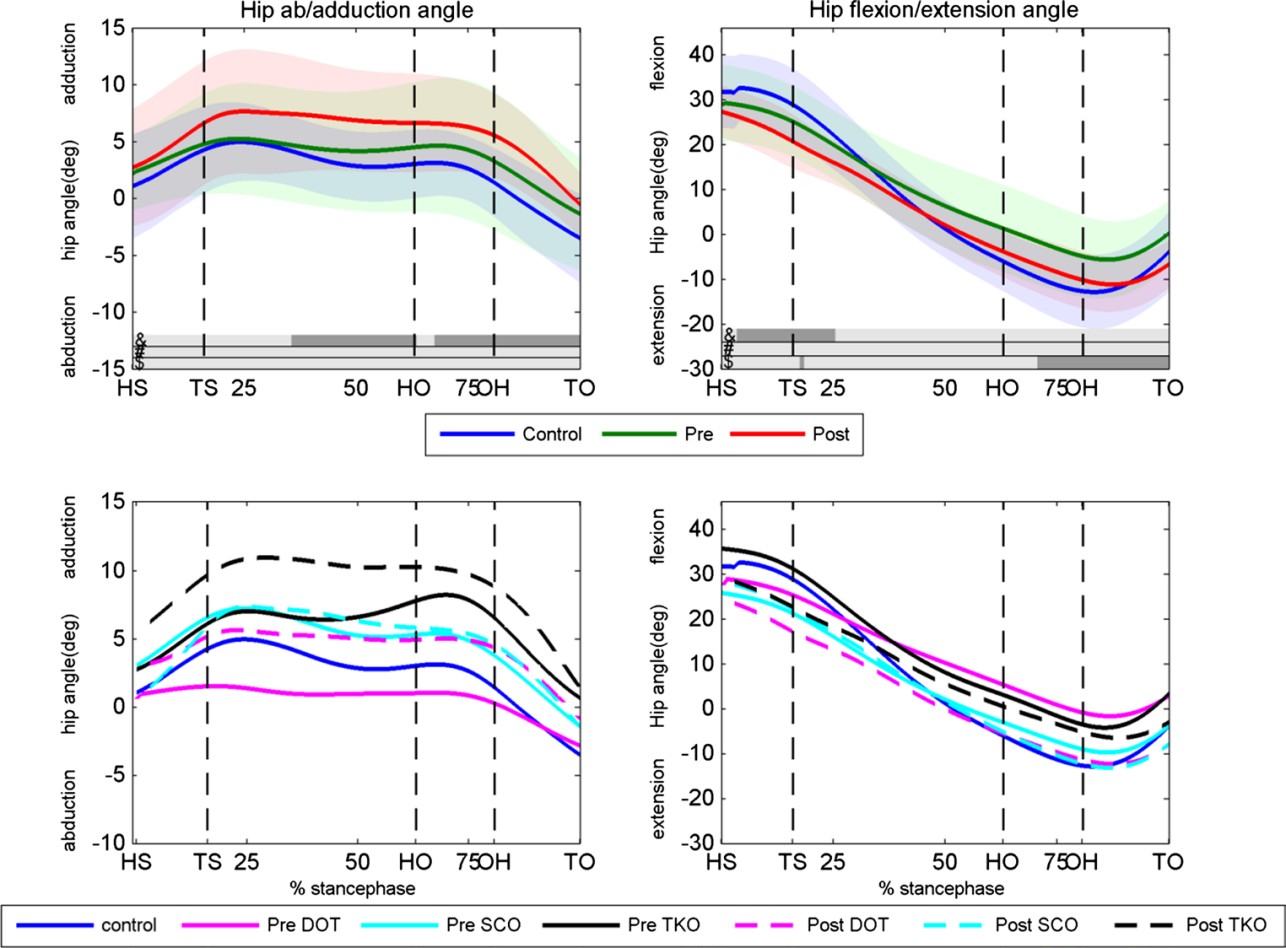
Hip abduction/adduction and flexion/extension angles. *Upper panels* the hip angles of the controls, preoperative condition and postoperative condition for the abduction/adduction (*left panel*) and flexion/extension (*right panel*) angle. Dark areas in the *bars right* above the *x*-axis indicate significant differences (*p* < 0.05) between: &, postoperative and controls, #, preoperative and controls, $, pre- and postoperative. *Lower panels* hip abduction/adduction and flexion/extension angles for the DOT, SCO and TKO group. Pre- and postoperative as well as the control data are displayed. *HS* heel strike, *TS* toe strike, *HO* heel off, *OH* opposite heel strike, *TO* toe-off, *Deg* degrees, *Pre* preoperative, *Post* postoperative, *DOT* double osteotomy, *SCO* supracondylar osteotomy, *TKO* high tibial osteotomy

There was no significant difference in hip flexion/extension angle between the preoperative condition and the controls (Fig. 3). After surgery, the hip flexion/extension angle was significantly lower at the final part of the stance phase compared to the preoperative condition. The patients had their hip significantly more extended at the first 25 % of the stance phase and more adducted from 35 to 100 % of the stance phase in the postoperative condition compared to the controls.

#### Knee and hip kinetics

**Fig. 4:**
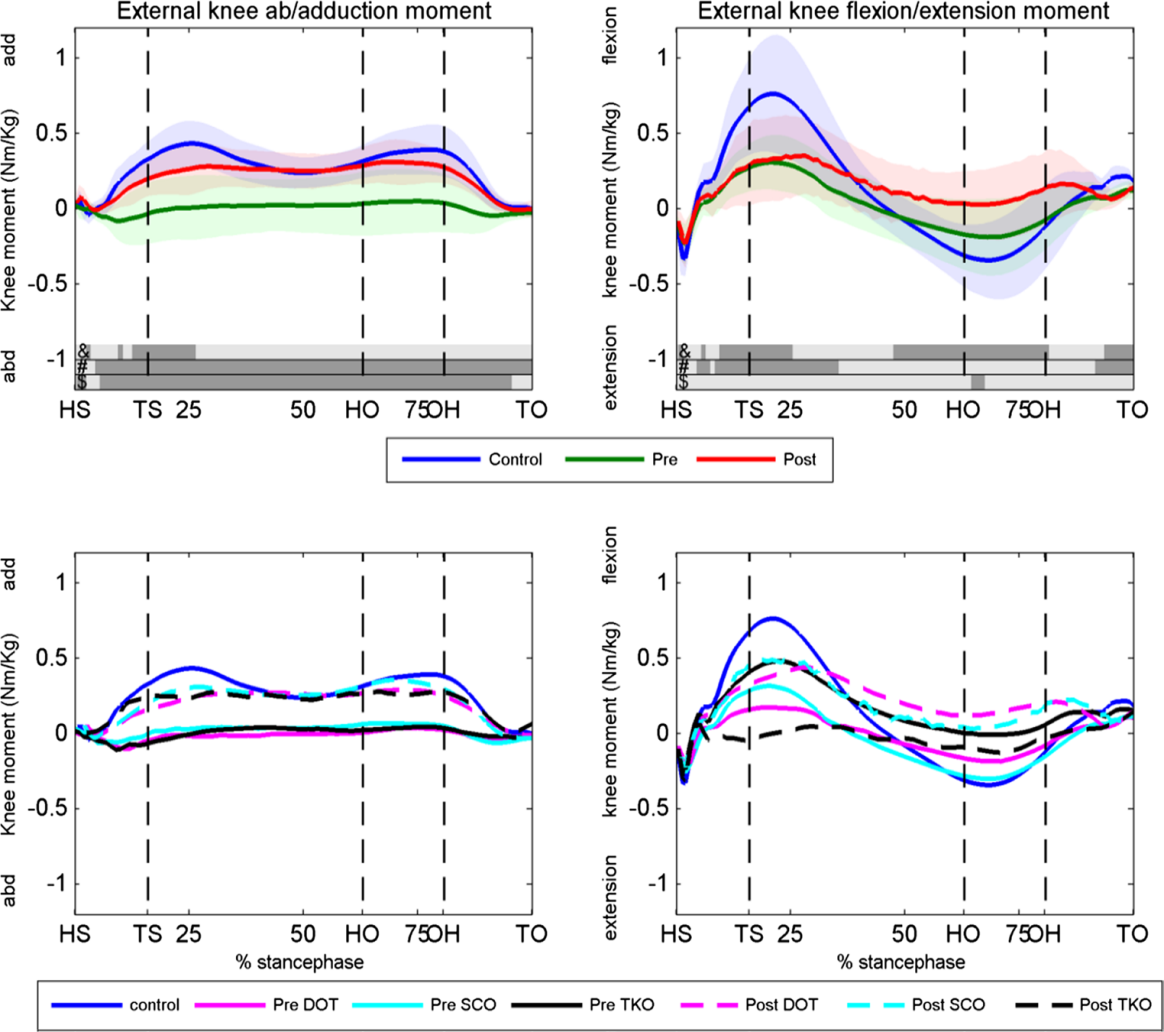
Knee external knee abduction/adduction and flexion/extension moments. *Upper panels* the knee moments of the controls, preoperative condition and postoperative condition for the abduction/adduction (*left panel*) and flexion/extension (*right panel*) moment. Dark areas in the *bars right* above the *x*-axis indicate significant differences (*p* < 0.05) between: &, postoperative and controls, #, preoperative and controls, $, pre- and postoperative. *Lower panels* knee abduction/adduction and flexion/extension moments for the DOT, SCO and TKO group and the controls. *HS* heel strike, *TS* toe strike, *HO* heel off, *OH* opposite heel strike, *TO* toe-off, *Deg* degrees, *Pre* preoperative, *Post* postoperative, *DOT* double osteotomy, *SCO* supracondylar osteotomy, *TKO* high tibial osteotomy

**Fig. 5:**
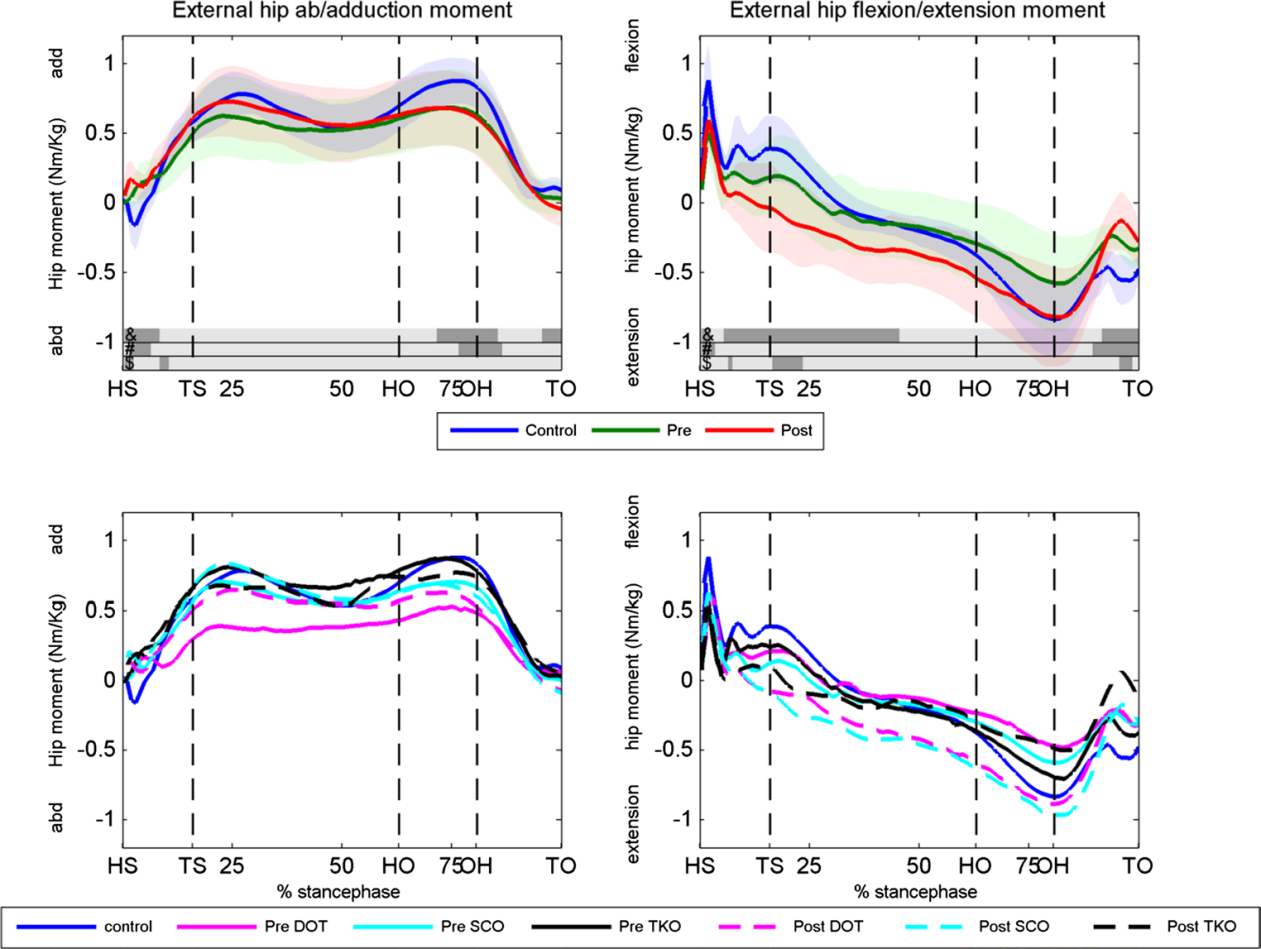
Hip abduction/adduction and flexion/extension moments. *Upper panels* the hip moments of the controls, preoperative condition and postoperative condition for the abduction/adduction (*left panel*) and flexion/extension (*right panel*) moment. Dark areas in the *bars right* above the *x*-axis indicate significant differences (*p* < 0.05) between: &, postoperative and controls, #, preoperative and controls, $, pre,- and postoperative. *Lower panels* hip abduction/adduction and flexion/extension moments for the DOT, SCO and TKO group and controls. *HS* heel strike, *TS* toe strike, *HO* heel off, *OH* opposite heel strike, *TO* toe-off, *Deg* degrees, *Pre* preoperative, *Post* postoperative, *DOT* double osteotomy, *SCO* supracondylar osteotomy, *TKO* high tibial osteotomy

The external knee and hip joint moments for the pre- and postoperative condition and the controls are shown in Figs. 4 and 5, respectively. The patients had a significant lower knee adduction moment before surgery compared to healthy controls, which increased significantly postoperative during almost the entire stance phase. The mean knee adduction moment increased significantly from 0.004 preoperative to 0.204 Nm/kg postoperative (*p* = 0.004). A power calculation based on the change in mean knee adduction moment revealed a power of 95.4 %. After surgery, patients had only a significant lower adduction moment compared to controls around toe strike. The patients had significantly lower knee flexion moment at the first 35 % of the stance phase and lower knee extension moment at toe-off (right upper panel, Fig. 4) compared to the controls. Surgery did not affect the knee flexion/extension moment.

Hip external abduction/adduction had almost no differences between the patients (pre- and postoperative) and the controls (Fig. 5). Surgery caused a significant decrease in hip extension moment around toe strike and toe-off. The hip extension moment was significantly lower after surgery at the first 50 % of stance and at toe-off compared to controls. No clear differences were found between the three surgical techniques in knee and hip moments (lower panels of Figs. 4, 5).

A significant correlation was found between the correction of HKA angle and increase in mean knee adduction moment (*r* = 0.65; *p* = 0.04).

## Discussion

The most important finding of the present study is the significant increase in knee adduction moment during the whole stance phase postoperatively to an almost similar pattern as was found in the control group (left upper panel Fig. 4). In addition to the increase in peak adduction moment (mean change was 0.72 % BW*Ht (95 % CI 0.42, 1.02) described by Collins et al. [6], an increased mean adduction moment during the whole stance phase of 0.20 Nm/kg after three types of osteotomies was found. Collins et al. [6] investigated the gait of a subgroup of 12 patients after a lateral opening wedge high tibial osteotomy for a mild valgus malalignment [mean HKA angle 2.4° (SD 2.4)]. The authors excluded patients with an abnormal mLDFA, and they did not compare the results to a control group. In contrast to Collins et al. [6], patients with a large HKA angle [mean HKA angle 9.3 (SD 5.7)] an abnormal mLDFA were included and compared to a control group. The knee adduction moment changed postoperatively during the whole stance phase to an almost similar pattern as the control group. Our control group showed comparable results with the gait characteristics of other healthy subjects in the literature [12, 35].

As yet to our knowledge only one gait study has been performed with varus osteotomies [6], the mechanical axis seems the best predictor of the peak abduction/adduction moment, as shown in earlier studies with valgus osteotomies [8, 14, 38, 39]. Turcot et al. [35] found that subjects with varus knees had larger peak knee adduction moments than subjects with neutral or valgus knees. A valgus osteotomy causes an increase in the abduction moment and a lateral shift in the dynamic knee joint loading [8, 36, 38, 39]. Postoperatively, a mean correction of 10.4° (95 % CI 6.4–14.4) towards a mean HKA angle of 1.1° (SD 2.3) varus and an increase in the adduction moment comparable to that of healthy controls during the entire stance phase were found. It seems that a varus osteotomy, which has an opposite effect as compared to a valgus osteotomy, caused a medial shift in the dynamic knee joint load. The medial shift will optimally restore cartilage loading forces and knee ligament balance and possibly reduces the risk of OA. The mean increase in knee adduction moment during stance showed a significant correlation with the correction of the valgus malalignment.

It was showed that the other gait kinetics and kinematics improved towards that of a healthy control group after surgery, with exception of the knee and hip flexion/extension motion and moment. In general, our patients showed less knee and hip flexion/extension motion and moment during gait compared to the controls. Postoperatively these curves hardly changed compared to preoperatively. The pre- and postoperative differences between the patients and the controls could be explained by the lower walking velocities, which were significantly different between the patients and the controls. Kirtley et al. [19] already showed that the peak knee flexion moment is strongly related to walking speed. Also Brinkmann and Perry [3] found a positive correlation between knee flexion and gait velocity.

Significant improvements in WOMAC, KOS, OKS and VAS-pm and VAS-pf were found in all patients. These clinical results are comparable with the literature, although there are a lot of discrepancies between studies and most studies have a low level of evidence [1, 2, 7, 10, 11, 30, 34]. The significant improvements of all clinical outcomes prove the effectiveness of a varus-producing osteotomy; however, long-term results are needed to confirm this conclusion.

A common limitation in studying patients with knee OA and a valgus alignment is the low prevalence of these patients [35]. In the current study, a small number of patients (12 in total) was evaluated. Nevertheless, the power appeared to be 0.95 for the difference in mean abduction moment. Another limitation is that three different operation techniques were used. However, different operation techniques are needed to maintain a normal knee joint line orientation after correction of each type of bone deformity. Maintaining a normal knee joint line orientation will optimally restore cartilage loading forces and knee ligament balance [2, 28] after correction, and this results in long-term survival of the osteotomies [2]. Although it was not possible to perform a statistical analysis of these three subgroups, the clinical evaluations, radiographic measurements as well as the kinetics and kinematics of gait were similar in all three operation techniques; therefore, the osteotomy type chosen does not seem to influence the outcome. The operated leg was compared to a control group, instead of the healthy leg. In most studies, the operated leg is compared to the healthy leg. However, the leg deformities in our study group were large, and compensatory mechanisms during gait could have been expected in the gait cycle of the contralateral leg. Therefore, the gait patterns of the operated leg were compared with that of a healthy control group in order to be able to compare it with a healthy gait cycle.

## Conclusion

This study showed that different types of varus-producing osteotomies in patients with lateral knee OA and a valgus alignment are a successful treatment in correcting alignment resulting in an increase in all postoperative clinical outcomes. A significant increase in the knee adduction moment was found during stance phase postoperatively, which was related to the degree of correction. Several other gait characteristics significantly changed towards that of the healthy controls. The HKA angle towards zero degrees caused a medial shift in the dynamic knee loading. The medial shift will optimally restore cartilage loading forces and knee ligament balance and reduces progression of OA or the risk of OA.
